# Facial Atrophy in Oral Submucous Fibrosis: An Association or a Coincidence

**DOI:** 10.1155/2016/2080467

**Published:** 2016-05-31

**Authors:** Sameep S. Shetty, Premalatha Shetty, Amruta Ramgonda Chougule

**Affiliations:** ^1^Department of Oral and Maxillofacial Surgery, Manipal College of Dental Sciences, Manipal University, Mangalore 575001, India; ^2^Department of Conservative Dentistry and Endodontics, Manipal College of Dental Sciences, Manipal University, Mangalore 575001, India

## Abstract

The anecdotal clinical presentation of OSMF that includes vesicle formation, burning sensation, intolerance to hot and spicy food, and trismus due to circumoral fibrous bands has been ringing in our ears for decades but the current paper flags novelty by portraying a rare presentation of an advanced stage of OSMF.

## 1. Introduction

The history of OSMF dates back to Sushrutha (2500–3000 BC) who had recognized it as a mouth and throat malady and coined it as “Vidhari” [1]. Areca nut, the main culprit behind it, is the fourth most addictive substance in the world [2] and is associated with a dependency syndrome. It has 600 million users in the world and is possibly the second most consumed carcinogen after tobacco in the Indian subcontinent [3]. Areca nut contains alkaloids, flavonoids, and copper which interfere with homeostasis of the extracellular matrix [4]. The characterization of its etiopathogenesis is debatable with no clear-cut consensus [5].

## 2. Case Report

**Figure 1:**
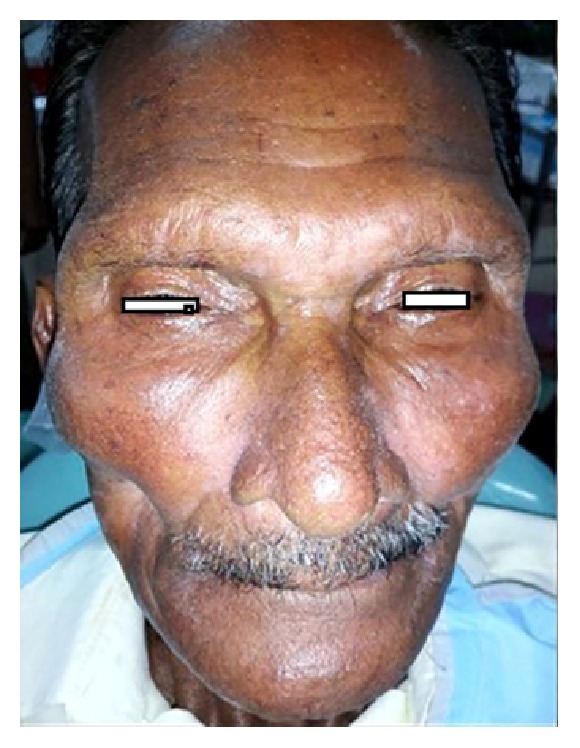
Bilateral facial atrophy seen.

**Figure 2:**
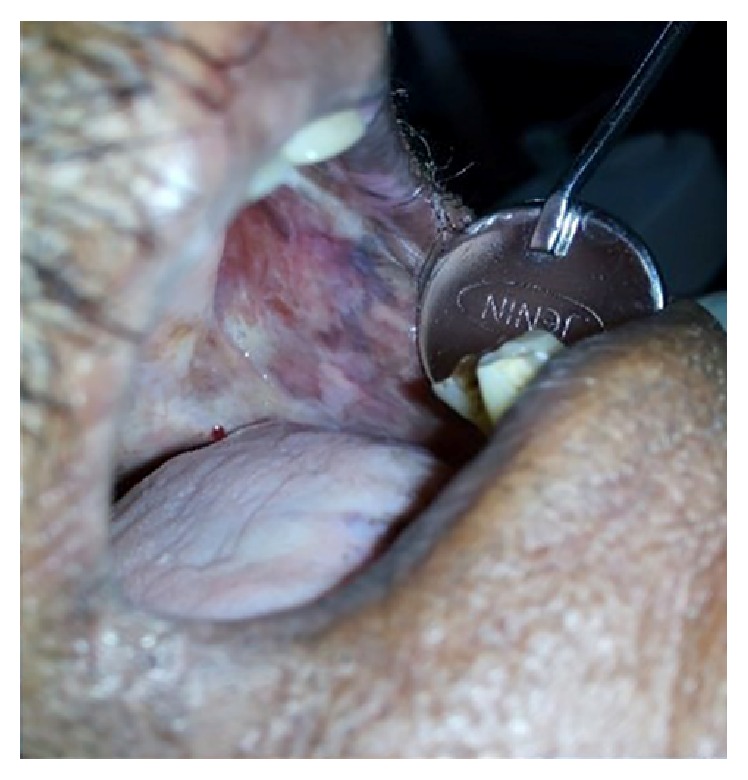
Blanching of the oral mucosa.

**Figure 3:**
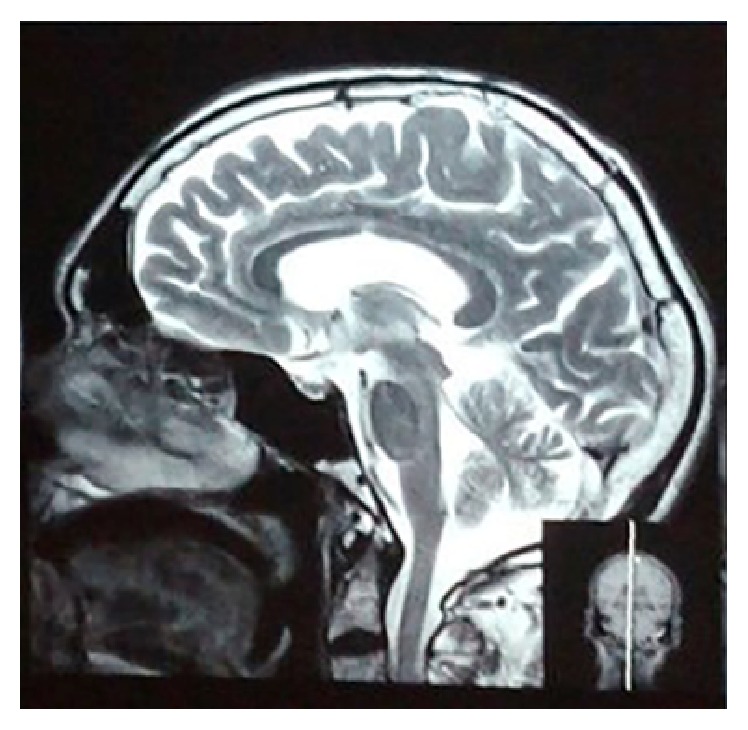
Brain MRI showing cerebral and cerebellar atrophy.

**Figure 4:**
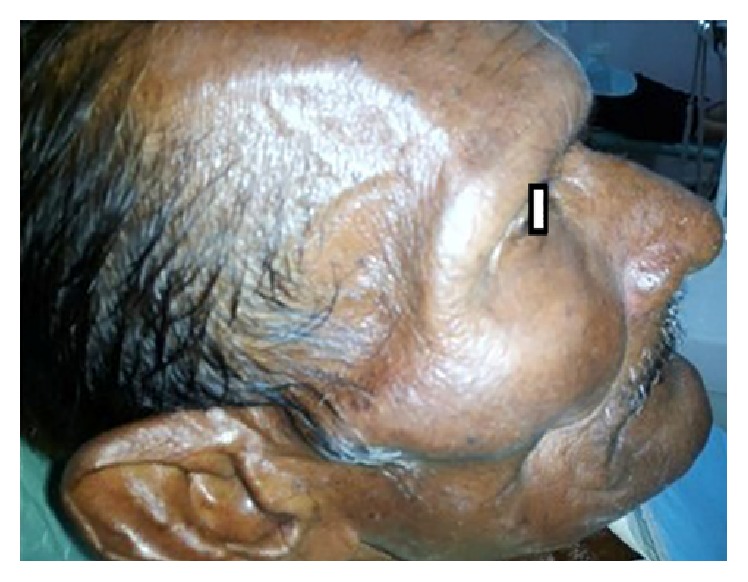
Profile view showing facial atrophy with loss of muscle and fat.

A 60-year-old man reported to the Department of Oral and Maxillofacial Surgery for total extraction. On clinical examination, extraorally, there was generalized bilateral facial atrophy with loss of muscle and fat (Figures 1 and 4). On detailed questioning, he disclosed history of chewing fresh areca nut 4-5 times a day for 20 years with symptoms of burning sensation, progressive restriction in mouth opening, and intolerance to hot and spicy food for 10 yrs. He was advised to go for surgical intervention but due to financial constraints he opted for conservative treatments that included use of antioxidants and intralesional injection of steroids; however, he discontinued the treatment abruptly and drastically limited his frequency of areca nut consumption. Intraoral examination revealed palpable bilateral fibrous bands on the buccal mucosa, thin lips, trismus of 3 cm (Figure 2), and absence of puffed cheek appearance while blowing. Recent bouts of sleeplessness for 1 yr compelled him to visit a neurologist who advised him to have brain MRI. To our surprise, MRI reported cerebral and cerebellum atrophy with ischemic changes (Figure 3). Incisional biopsy of the buccal mucosa showed OSMF with moderately advanced epithelial dysplasia.

## 3. Discussion

Areca nut is the fourth most commonly used psychoactive substance chewed as an aid to digestion and as a stimulant, either used alone or flavored with different tobacco or nontobacco substances. Areca nut contains several alkaloids, of which arecoline is the most abundant. Arecoline is mutagenic in both bacteria and mammalian cells [6]. Arecoline forms at least four nitrosamines upon nitration; these products include N-nitrosoguvacine, N-nitroguvacoline, 3-(methylnitrosamino) propionitrile (MNPN), a potent carcinogen, and 3-(methylnitrosamino) propionaldehyde (MNPA) and two unknown N-nitrosamines [7].

Areca nut apart from being carcinogenic has diverse effects on almost all organs of the human body including the brain, heart, lungs, gastrointestinal tract, and reproductive organs. It interferes with fat metabolism leading to type 2 diabetes, metabolic syndromes, and deranged blood lipid levels. The alkaloids in areca nut, namely, arecoline, arecaidine, guvacoline, and guvacine, have powerful parasympathetic and muscarinic effects which produce euphoria and counteract fatigue [8, 9]. It also activates sympathoadrenal response. Areca nut has a direct antimicrobial effect against bacteria, including* Streptococcus mutans*,* Streptococcus salivarius*, and various other microorganisms in the oral cavity. The cariostatic properties of areca nut shield its users from dental caries; however, they commonly fall prey to periodontal infections [10].

Overactivity of the masticatory muscles results in excess glycogen consumption and the diminished blood supply following fibrotic changes of the connective tissue leads to muscle degeneration [11].

OSMF is amenable to primary prevention and needs to be stressed upon in view of the lack of availability of curative treatment. The euphoria and energizing action associated with betel quid seems to boost the endurance of a person leading to habitual consumption; thus, curtailing its progress seems to be a herculean task. Clearly, further studies are warranted to elicit the differential systemic effects of areca nut before we arrive at our final noteworthy conclusion of facial atrophy associated with oral submucous fibrosis.

## References

[1] Gupta S. C., Yadav Y. C. (1978). ‘Misi’ an etiologic factor in oral submucous fibrosis. *Indian Journal of Otolaryngology*.

[2] Naik S. M., Naik S. S. (2012). A study of 63 cases of mouth neoplasms in arecanut growing belt of Sullia. *Iranian Journal of Cancer Prevention*.

[3] Joseph N., Nagaraj K., Shashidhar Kotian M. (2010). Areca nut and tobacco use among school children in a village in South India—a cross sectional study. *Australasian Medical Journal*.

[4] Arakeri G., Brennan P. A. (2013). Oral submucous fibrosis: an overview of the aetiology, pathogenesis, classification, and principles of management. *British Journal of Oral and Maxillofacial Surgery*.

[5] Aziz S. R. (2008). Oral submucous fibrosis: case report and review of diagnosis and treatment. *Journal of Oral and Maxillofacial Surgery*.

[6] Shirname L. P., Menon M. M., Nair J., Bhide S. V. (1983). Correlation of mutagenicity and tumorigenicity of betel quid and its ingredients. *Nutrition and Cancer*.

[7] Shwetha H. R., Babu C. N., Ahmed S., Garg A. (2015). Effect of arecanut on oral epithelium—a review of literature. *University Journal of Dental Sciences*.

[8] Garg A., Chaturvedi P., Gupta P. C. (2014). A review of the systemic adverse effects of areca nut or betel nut. *Indian Journal of Medical and Paediatric Oncology*.

[9] Strickland S. S., Duffield A. E. (1998). Nutrition and ecosystems in Sarawak: the role of the areca nut. *Asia Pacific Journal of Clinical Nutrition*.

[10] Lingappa A., Napalli D., Sujatha G. P., Shiva Prasad S. (2011). Arecanut: to chew or not to chew?. *e. Journal of Dentistry*.

[11] Khanna J. N., Andrade N. N. (1995). Oral submucous fibrosis: a new concept in surgical management. Report of 100 cases. *International Journal of Oral and Maxillofacial Surgery*.

